# Geological and climatic influences on natural radioactivity in drinking water and their health impacts: a study of Dessie and Kombolcha towns, Ethiopia

**DOI:** 10.1038/s41598-026-43834-9

**Published:** 2026-03-17

**Authors:** Hailu Geremew, Yimam Mekonnen, Ashenafi Admasu

**Affiliations:** Department of Physics, Wollo University, P. O. Box 1145, Dessie, Ethiopia

**Keywords:** Natural radioactivity, Uranium and thorium concentrations, Geochemical factors, Radiation dose assessment, Health risks in Ethiopia, Climate sciences, Environmental sciences, Hydrology, Natural hazards

## Abstract

This study examines the geological and climatic factors that influence natural radioactivity levels in drinking water from Dessie and Kombolcha towns in Ethiopia’s northern plateau. The basaltic and sedimentary mudrock formations of the region contain high levels of natural radionuclide elevated natural radionuclide levels, while climatic factors such as precipitation, soil moisture, and humidity enhance their mobility and transport into water sources. Kombolcha, situated at a Lower altitude geographically, exhibits higher radionuclide concentrations for ^238^U and ^232^Th. All measured values are almost greater than the global permissible values 0.5 mBq/L and 0.05 mBq/L respectively, especially for samples collected from running water. This is due to surplus and downward transport along the Borkena River from Dessie to Kombolcha, which is intensified by climatic conditions and rainfall. ^40^K shows high activity values in samples from underground and spring water in both towns, even if its global permissible value is clearly not known in ingestion food type. Uranium’s greater solubility compared to thorium results in higher activity levels above 0.5 mBq/L, across both towns in this study. Radiation dose assessments reveal significant health risks, particularly in spring and headwater areas. Annual effective doses exceed the permissible values recommended by international guidelines like IAEA and ICRP in both towns which is 1 mSv/yr. These findings suggest potential long-term health risks, with recommendations for continuous monitoring and mitigation measures to protect public health in the two towns and downstream from the Ethiopian northern plateau.

## Introduction

Radionuclides such as uranium, thorium, and potassium-40 are of natural origin and occur within various rock types in the Earth’s crust. These elements can leach into water sources over time, a process governed by the specific geochemical conditions of the environment. Consequently, the geological formation and climatic condition of a region significantly influences the concentration and spatial distribution of these radionuclides in drinking water^1,2^.

Uranium commonly occurs in granitic and sedimentary lithologies containing uraninite, pitchblende, and coffinite, and these geological settings often exhibit elevated groundwater uranium concentrations. Its mobility is largely controlled by redox state and bicarbonate-rich conditions, which promote formation of soluble uranyl-carbonate complexes^3–5^. Research consistently demonstrates that regions dominated by these lithologies exhibit significantly higher uranium concentrations in comparison to other environmental settings. This enrichment facilitates the natural leaching of uranium into groundwater, a process dictated by its solubility behavior under specific geochemical conditions, such as bicarbonate content and redox potential. For instance, studies in Finland have shown that groundwater in granitic bedrock areas possesses markedly higher uranium levels reaching concentrations as high as 20,000 µg/L than groundwater in other geological terrains^6,7^.

Thorium occurs primarily within igneous and metamorphic rocks, specifically in thorium-rich minerals such as monazite and thorite. Investigations indicate that thorium concentrations in drinking water are generally lower than uranium owing to its limited solubility in groundwater^8^. Nevertheless, elevated thorium levels have been reported in groundwater situated within thorium-rich geological formations, such as those found in parts of India and Western Africa^2,7,9^.

Recent hydrogeochemical studies confirm that potassium in groundwater is largely derived from the weathering of silicate minerals such as feldspar and mica in volcanic and crystalline rocks, with volcanic terrains often exhibiting enhanced mineral dissolution and groundwater mineralization^10,11^. Although potassium is an essential nutrient for human physiological development, research in regions such as Japan has demonstrated that areas characterized by volcanic rocks exhibit significantly higher concentrations of ^40^K in groundwater^2,12^.

Drinking water contaminated with natural radionuclides poses significant health risks. Uranium-238 in drinking water is of particular concern because of its combined chemical toxicity and radioactivity. Chronic ingestion has been associated with nephrotoxicity due to uranium accumulation in the kidneys, while inhalation or internal exposure to uranium decay progeny may increase lung cancer risk^13–15^. Furthermore, uranium’s radiological hazard twigs from its decay products, which emit alpha particles. Radon, a decay product of uranium, is a significant concern in drinking water. It can be released into the air during water use, contributing to indoor air pollution. Inhalation of radon and its progeny increase the risk of lung cancer^16,17^.

Potassium (^40^ K) is an essential nutrient; however, its radioactive isotope contributes to the internal radiation dose received by humans through ingestion pathways^18^. The health risks associated with ^40^ K are generally lower compared to uranium and thorium, due to its widespread distribution and essential biological role in human’s body^19^.

The towns of Dessie and Kombolcha, located on the northern Ethiopian plateau, are rapidly growing urban centers that rely on diverse water sources, including rivers, springs, and groundwater. Despite this reliance, the potential for radionuclide contamination in drinking water, as well as the influence of geological and climatic factors on these towns and their downstream areas, has not been adequately studied. In this study, sources of natural radionuclides, the geologies of study area and factors for mobilities of natural radionuclides, the climatic conditions are considered to measure activity concentrations of natural radionuclides in drinking water. The geology of the study area is characterized by high-altitude volcanic formations and is underlain by radionuclide-bearing rocks, including sediments and basalts. This geological setting and climatic conditions underscore the need for a systematic investigation of activity concentration in drinking water to ensure the safety and health of populations dependent on water sources. The main objective of this study is to assess and analyze levels of natural radioactivity in drinking water, specifically concentrations of uranium, thorium, and potassium, arising from geological and climatic conditions. In addition, the study evaluates the associated radiological health risks in Kombolcha and Dessie using gamma-ray spectrometry. As with any field-based investigation, limitations related to sample size and representativeness may occur. Moreover, the study is based on data collected over one year, July 2024 to June 2025, highlighting the need for continuous monitoring with a larger dataset to capture temporal variations.

## Materials and methods

This study integrates geological and climatic analyses with radiometric measurements to examine the factors influencing natural radioactivity in drinking water. Water samples were collected from the towns of Dessie and Kombolcha, which represent distinct altitudinal and geological settings. The methodology involved gamma spectrometry to measure radionuclide activity, geological characterization of formations surrounding the water sources, and the integration of climatic parameters, including precipitation, humidity, and soil moisture. Climatic data for the study period from 2020 to 2022 were obtained from NASA POWER datasets and used to assess the annual mobility of radionuclides.

### The study areas

The study samples were collected from the towns of Dessie and Kombolcha, which are located at elevations ranging from 2470 to 2550 m above sea level. Both towns are characterized by abundant water resources, including groundwater, spring water, and river systems. The study area covers 26 km, following the river flow from Dessie to Kombolcha. These two towns have experienced rapid urbanization in recent years and host one of Ethiopia’s key industrial zones.

**Fig. 1:**
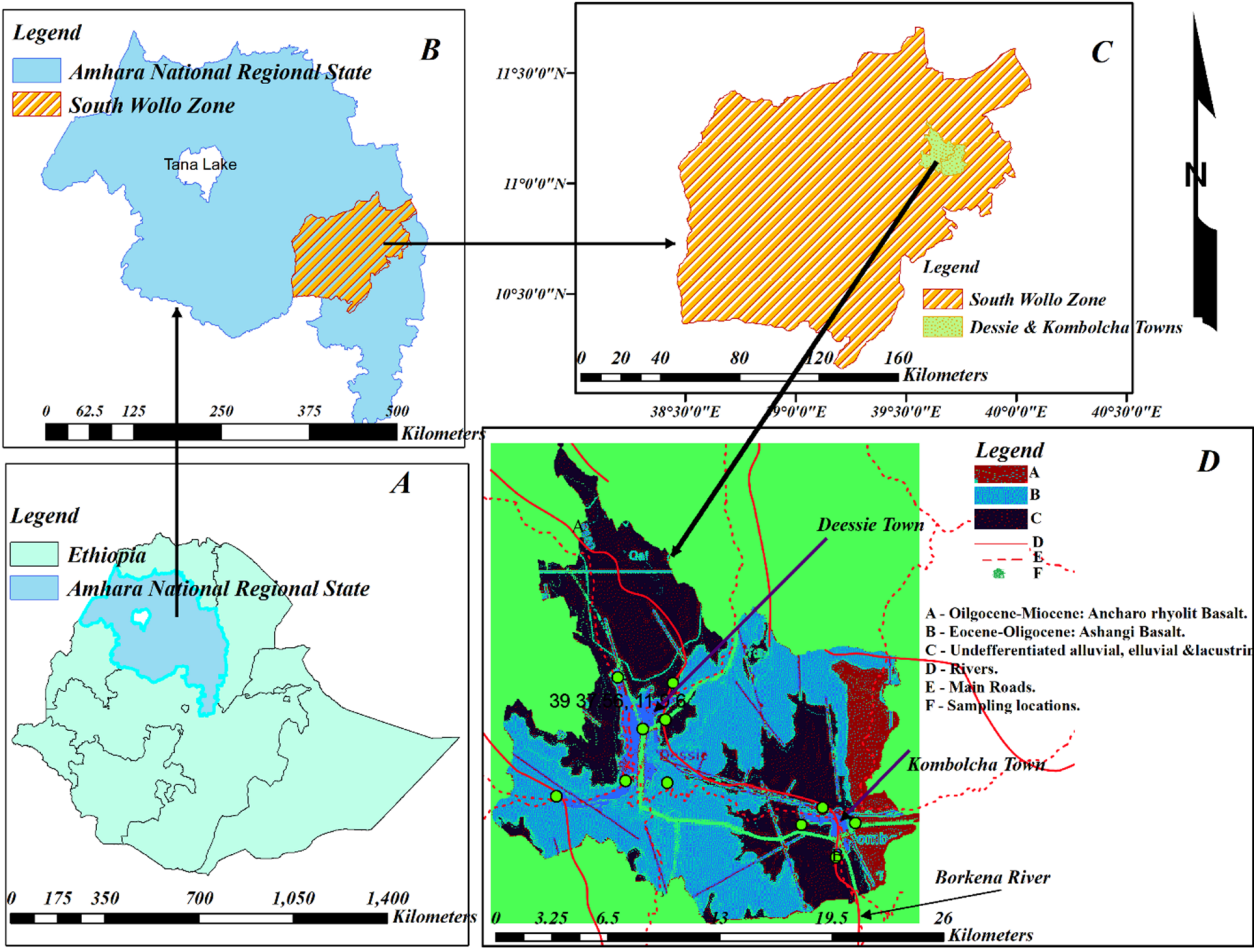
Geographical location, geological formations, and climatic context (altitude, rainfall patterns) of Dassie and Kombolcha towns.

Figure 1 was developed using ArcGIS version 10.7.1. and part D was adopted from tiff file developed by Ethiopian Geological Survey Agency. As shown in the figure, the area is composed of diverse basaltic and sedimentary rock units. These lithologies host naturally occurring radionuclides at varying concentrations and were taken into account in this study.

### Sample collection and preparation

A purposive sampling approach was employed across the two study areas. Groundwater and spring water were sampled at their respective sources, while running water samples were obtained at multiple points along the flow gradient, ranging from headwaters (upstream) to downstream reaches. The data collection and measurement were conducted over a 12-month period, from July 2024 to June 2025. Sampling was conducted using standardized procedures recommended by national and international guidelines to minimize the risk of contamination. For each sampling site, one liter (1 L) of water was collected, and 0.1 mL of hydrochloric acid (HCl) was added to each sample to inhibit microbial growth on the walls of the airtight plastic containers^20^. The samples were then stored for a minimum of 28 days to allow secular equilibrium to be established, ensuring uniform decay between parent radionuclides and their daughter products. After this period, each sample was measured for more than 10 hours to obtain radionuclide spectra with sufficient statistical significance for analysis^21^.

### Principle of gamma spectrometry

Sodium Iodide based gamma spectrometry is a technique used to measure and analyze the energy and intensity of gamma rays emitted by radionuclides found in drinking waters collected from the study areas. Sodium iodide (NaI) detectors are commonly used due to their high efficiency. For the purpose of this study, we used a moderate sized NaI(Tl), thallium activated detector of 4.5 cm by 4.5 cm crystal size. After photomultiplier tube, we used MASTRO-32 computer program to background measurement and analyze gamma spectrum for each sample.

### Equipment and calibration

The gamma spectrometry system consists of all the necessary components for accurate measurements. Radiation shielding was achieved using a compacted 10 cm thick concrete enclosure laminated with thin aluminum foil to reduce gamma-ray backscattering. Proper calibration of the spectrometer is essential for reliable measurements and was carried out using standard gamma-emitting radionuclide sources, Cs-137 and Na-22, to establish the detector’s energy calibration and efficiency. The calibration utilized the 661.7 keV gamma line of Cs-137 and the 511 keV and 1274 keV gamma lines of Na-22. The detector efficiency was determined to be 8% at 661.7 keV using the Cs-137 standard source.

The energy resolution of the detector was evaluated using the ratios of the full width at tenth maximum (FWTM) to the full width at half maximum (FWHM), and the full width at fiftieth maximum (FWFM) to the FWHM, based on the 661.7 keV photopeak of the Cs-137 check source. The obtained ratios were less than 1.9 and 2.5, respectively, indicating good energy resolution and effective discrimination of the photopeak from nearby peaks and background radiation^22^.

### Measurement and analysis

The prepared samples were placed in the NaI detector, and gamma spectra were acquired over a specified counting period of at least 10 h to ensure adequate counting statistics. Prior to sample measurement, an empty sample container was placed on the detector to measure background radiation for approximately the same duration as the sample measurements. This background measurement was repeated midway through the sample measurements to check for possible variations in background radiation. After all samples were measured, the average background values were subtracted from each sample spectrum.

For some samples, the measured activity was comparable to the corresponding background values. These results were therefore considered to be below the detection limit (BDL), with the minimum detectable value (MDV) being close to zero. In the majority of measurements, the activity concentrations of the samples were higher than those of the background radiation. These spectra were subsequently analyzed to identify and quantify the radionuclides present in the water samples, expressed per liter, using the MASTRO-32 computer program. The activity concentrations of the identified radionuclides were then calculated using the following formula:

1 $$A=\frac{C}{E.t.V}$$

where A is the activity concentration (Bq/L), C is the net count rate (counts per second), E is the detection efficiency, t is the counting time (seconds), V is the volume of the sample (liters).

### Quality control and assurance

Quality control measures include the use of blank samples for the reduction of background radiation, duplicate samples that were collected from different site, and standard reference materials, soil-6 and soil-375 to ensure the accuracy and reliability of the results. Regular calibration of the gamma spectrometry system and validation of analytical procedures were considered for the quality assurance^23^.

### Data interpretation and radiation dose calculation

The obtained activity concentrations of radionuclides will be used to calculate the annual effective dose for individuals consuming the water^24–26^. This will be done using the dose conversion factors provided by the International Commission on Radiological Protection (ICRP) as follows:

2 $$D=\sum_{i=1}^{n}Ci\left(D.C.{F}_{i}.I\right)$$

where D is the annual effective dose (mSv/year), C_i_ is the activity concentration of radionuclide i (Bq/L), DCF_i_ is the dose conversion factor for radionuclide i (mSv/Bq), I is the annual intake of drinking water (730 L/year for adult).

*DCF*_*i*_ for ^238^U is 4.5 × 10^-5^ mSv/Bq, for ^232^Th is 2.3 × 10^-4^ mSv/Bq and for ^40^K is 6.2 × 10^-6^ mSv/Bq ^27^*.*

## Results and discussions

### Results

**Fig. 2:**
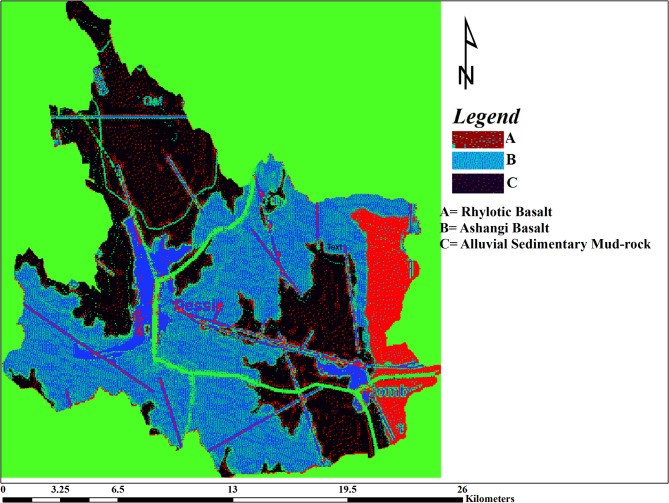
Geological Spatial distribution of the study area.

Gamma spectrometry analysis of drinking water samples from Dessie and Kombolcha revealed variations in natural radionuclide activity concentrations that correlate closely with both geological formations and climatic conditions. Water sources situated within basaltic and sedimentary mud-rock formations exhibited elevated concentrations of uranium and thorium as seen from Fig. 2. As represented in the figure by blue color or letter B, the area is aquatic enriched by spring water and runway of the rivers. Furthermore, annual precipitation and soil moisture appear to facilitate the leaching and hydrogeochemical transport of these radionuclides and their decay progeny.

The geochemical characteristics of these formations are influenced by annual climatic conditions, which in turn affect the mobility and distribution of the radionuclides. Kombolcha, located at a lower elevation, consistently demonstrated higher activity levels than Dessie as it is observed in below tables, reflecting both its geological setting and the influence of meteoric runoff and fluvial transport. Overall, the greater solubility and mobility of uranium relative to thorium accounts for its consistently higher activity levels across the sampled water sources.

#### Natural radionuclide activity concentration

The samples were classified into three categories: groundwater used for drinking, head or spring water, and running or river water. A purposive sampling technique was employed, taking into account the drainage patterns of water sources within the sub-study areas. Three samples were collected from each sub-study area, and their activity concentrations were measured. Finally, the average activity concentrations of ^232^Th, ^238^U, and ^40^ K were calculated to reduce measurement uncertainty, following the procedures described in Sections "Principle of gamma spectrometry", "Equipment and calibration", and "Measurement and analysis".

Table 1 summarizes the results of natural radioactivity concentrations in underground drinking water collected from the capital of the South Wollo Zone and the industrial town, Kombolcha. The measurements indicate that the thorium activity concentrations in samples collected from Dessie town, Gerado, and Tita are below the permissible values suggested by a joint report of the IAEA and WHO^28^. However, the Boru site shows a slightly higher value than the permissible limit for thorium. The uranium activity concentration in the same sampling area exceeds the recommended value from the report. Samples from Kombolcha town exceed the permissible values for both thorium and uranium activity concentrations. Potassium-40, as labeled and explained in Table 1, has no permissible limit due to its importance for the human body.

**Table 1:** Activity concentration of natural radionuclides in underground drinking water and their relation to local geological formations and climatic influences.

Selected Towns	Site/Station	Sample Code	^Activity concentration (mBq/L)^		
			^232^Th	^238^U	^40^K
Dessie	Gerado	S1	0.038 ± 0.005	1.87 ± 0.14	131 ± 3
		S2	BDL	BDL	320 ± 5
		S3	0.079 ± 0.002	1.52 ± 0.06	123 ± 2
		Avg	0.039 ± 0.005	1.13 ± 0.15	191 ± 6
	Tita	S1	BDL	1.19 ± 0.18	108 ± 2
		S2	0.089 ± 0.008	BDL	430 ± 7
		S3	BDL	0.93 ± 0.08	292 ± 4
		Avg	0.030 ± 0.008	0.71 ± 0.20	276 ± 9
	Boru	S1	0.080 ± 0.014	1.89 ± 0.21	260 ± 5
		S2	0.051 ± 0.006	0.72 ± 0.03	560 ± 5
		S3	0.034 ± 0.012	1.05 ± 0.11	389 ± 4
		Avg	0.055 ± 0.019	1.23 ± 0.24	403 ± 7
Kombolcha	Station 1	S1	0.090 ± 0.016	1.15 ± 0.19	108 ± 2
		S2	0.070 ± 0.009	1.43 ± 0.02	430 ± 7
		S3	0.087 ± 0.015	0.96 ± 0.08	292 ± 4
		Avg	0.082 ± 0.016	1.18 ± 0.21	277 ± 9
	Station 2	S1	0.064 ± 0.011	1.69 ± 0.22	179 ± 3
		S2	0.078 ± 0.012	0.52 ± 0.03	560 ± 5
		S3	0.044 ± 0.010	1.12 ± 0.11	389 ± 4
		Avg	0.062 ± 0.019	1.11 ± 0.25	276 ± 6
Reference Values^28^			0.05	0.5	*

* ^40^K, a radionuclide that occurs naturally in a fixed ratio to stable potassium, is not included. This is because potassium is an essential element for humans and its concentration in the body is controlled by metabolic processes, BDL is for Below Detection Limit.

For head/spring water, samples were collected from three springs (Mume, Sireminch, and Minchitu) used for drinking and other purposes in Dessie town, and two springs (Station 1 and Station 2) in Kombolcha town. Three samples were prepared and analyzed for each head/spring water source as they were for underground water samples. Table 2 shows the experimental results of natural radioactivity concentrations in head/spring water. The experimental results indicate that the natural radionuclides have almost the same properties, with thorium activity concentrations being less than uranium activity concentrations in all samples from both study areas. However, the activity concentrations for head/spring water samples are higher than those for underground water for the three natural radionuclides.

**Table 2:** Activity concentration of radionuclides in head (spring) water, highlighting geological sources and climate-driven mobilities.

Selected Towns	Site/Station	Sample Code	^Activity concentration (mBq/L)^		
			^232^Th	^232^U	^40^ K
Dessie	Mume	S1	0.08 ± 0.03	1.8 ± 0.4	333 ± 4
		S2	BDL	1.36 ± 0.12	620 ± 5
		S3	0.14 ± 0.05	BDL	280 ± 2
		Avg	0.07 ± 0.06	1.05 ± 0.46	411 ± 4
	Sireminch	S1	0.071 ± 0.005	2.12 ± 0.15	741 ± 6
		S2	0.057 ± 0.011	0.78 ± 0.06	518 ± 5
		S3	0.023 ± 0.009	BDL	846 ± 8
		Avg	0.051 ± 0.015	0.97 ± 0.07	702 ± 6
	Minchitu	S1	0.06 ± 0.03	1.77 ± 0.20	BDL
		S2	BDL	2.3 ± 0.5	290 ± 2
		S3	BDL	BDL	473 ± 3
		Avg	0.02 ± 0.03	1.35 ± 0.56	188 ± 2
Kombolcha	Station 1	S1	0.021 ± 0.004	2.21 ± 0.16	646 ± 6
		S2	0.087 ± 0.021	1.71 ± 0.08	432 ± 5
		S3	0.043 ± 0.010	1.21 ± 0.06	567 ± 6
		Avg	0.050 ± 0.004	1.71 ± 0.16	548 ± 10
	Station 2	S1	0.02 ± 0.03	3.7 ± 0.8	363 ± 3
		S2	0.063 ± 0.018	2.5 ± 0.8	392 ± 3
		S3	0.043 ± 0.008	3.3 ± 0.6	543 ± 4
		Avg	0.04 ± 0.03	3.1 ± 1.3	433 ± 6
Reference Values ^28^			0.05	0.5	*

* ^40^K, a radionuclide that occurs naturally in a fixed ratio to stable potassium, is not included. This is because potassium is an essential element for humans and its concentration in the body is controlled by metabolic processes, BDL is for Below Detection Limit.

The activity concentration of thorium in spring water from Dessie and Kombolcha towns is nearly equal to the recommended value of 0.05 mBq/L, as reported jointly by the FAO, WHO, and IAEA ^28^. However, the uranium activity concentration in the same water samples exceeds the recommended value of 0.5 mBq/L. Additionally, Potassium-40 shows elevated activity concentrations in both towns. This radionuclide is categorized as a light nuclide, and most light nuclides are utilized in body development. Therefore, the radiation dose from Potassium-40 is not considered as significant as that from heavy nuclides^28^. It is important to note that the results vary across different locations due to the geological and geochemical characteristics of the headwaters. The measured activity concentrations of the three radionuclides are higher in the headwaters from Dessie compared to those from Kombolcha. This difference is attributed to the distinct geological and topographical features of the two towns, which affect the chemical properties of the radionuclides.

Samples of running water were collected from two rivers in each town: the Borkena and Gerado rivers in Dessie, and the Borkena and Berberewonze rivers in Kombolcha. Notably, the Borkena River flows through both towns, while the Gerado River is confined to Dessie. Three representative samples were taken from each river, and the average value was used for subsequent analysis. Table 3 presents the experimental results of natural radioactivity concentration in the running water, measured in milli becquerels per liter (mBq/L). The mean activity concentrations in the river water samples exceeded those found in underground and headwater samples. It is important to note that the water was unfiltered during sampling; untreated river water samples were analyzed directly.

**Table 3:** Activity concentration of radionuclides in running water, showing the combined effects of geological background and precipitation-driven runoff.

Selected Towns	Site/Station	Sample Code	^Activity concentration (mBq/L)^		
			^232^Th	^232^U	^40^K
Dessie	Borkena	S1	0.054 ± 0.012	0.78 ± 0.03	161 ± 7
		S2	0.18 ± 0.06	1.41 ± 0.15	225 ± 9
		S3	BDL	1.02 ± 0.12	89 ± 1
		Avg	0.08 ± 0.06	1.07 ± 0.19	158 ± 6
	Gerado	S1	0.10 ± 0.03	1.32 ± 0.07	128 ± 4
		S2	BDL	0.55 ± 0.07	130 ± 2
		S3	0.19 ± 0.07	0.18 ± 0.01	101 ± 2
		Avg	0.10 ± 0.07	0.69 ± 0.07	86 ± 2
Kombolcha	Berberewonze	S1	0.04 ± 0.02	0.78 ± 0.03	161 ± 7
		S2	0.15 ± 0.08	1.41 ± 0.15	225 ± 9
		S3	0.05 ± 0.04	1.02 ± 0.12	89 ± 1
		Avg	0.08 ± 0.09	1.07 ± 0.12	158 ± 1
	Borkena	S1	0.069 ± 0.013	1.31 ± 0.07	383 ± 8
		S2	0.076 ± 0.014	1.51 ± 0.07	418 ± 9
		S3	0.27 ± 0.09	2.13 ± 0.01	220 ± 3
		Avg	0.14 ± 0.09	1.65 ± 0.10	340 ± 6
Reference Values ^28^			0.05	0.5	*

* ^40^K, a radionuclide that occurs naturally in a fixed ratio to stable potassium, is not included. This is because potassium is an essential element for humans and its concentration in the body is controlled by metabolic processes, BDL is for Below Detection Limit.

If the river that crosses the two town (Borkena) considered, activity concentration at Kombolcha town is higher than at Dessie town. The flow of rainfall and other geochemical solutions are from Dessie to Kombolcha town. This can be considered as a cause of variation of activity concentration at the two towns. The more soluble radionuclide (Uranium) has more concentration in the river water. Geological and geographical variation of activity concentration of potassium does not see in the measurement as thorium and uranium. This can be as a result of chemical properties of potassium hosting geological formations.

#### Radiation doses from natural radionuclides in drinking water

Numerous studies have investigated the occurrence of natural radionuclides in drinking water from various sources, including groundwater, surface water, and bottled water. The concentrations of these radionuclides vary significantly depending on the geological characteristics of the region, water treatment processes, and other factors. Based on the investigated activity concentrations on natural radionuclides in underground water samples, the resulting radiation dose for the selected towns according to Eq. 2 are as in table below (Table 4).

**Table 4:** Average activity concentration and annual effective dose from radionuclides in underground drinking water, linked to geological formations and climate factors.

Selected Towns	Site or Station	^Average Activity concentrations (mBq/L)^			Annual effective dose (mSv/year),
		^232^Th	^238^U	^40^K	
Dessie	Gerado	0.039 $\pm$ 0.005	$1.130\pm$ 0.152	$191\pm 6.451$	0.86
	Tita	0.030 $\pm 0.008$	$0.706\pm 0.198$	$276\pm 8.921$	1.25
	Boru	0.055 $\pm 0.019$	$1.230\pm 0.239$	$403\pm 7.301$	1.82
Kombolcha	Station 1	0.082 $\pm 0.016$	1.179 $\pm 0.209$	277 $\pm 8.921$	1.25
	Station 2	0.062 $\pm 0.019$	1.112 $\pm 0.246$	276 $\pm 6.281$	1.25
Reference Values^28^		0.05	0.5	*	1

* ^40^K, a radionuclide that occurs naturally in a fixed ratio to stable potassium, is not included. This is because potassium is an essential element for humans and its concentration in the body is controlled by metabolic processes.

Annual effective dose in millisievert per year; exceed the reference annual effective dose of 1 mSv/year except Gerado site. Boru site (in Dessie town) shows the highest value of 1.82 mSv/year. Activity concentrations of the three radionuclides at this site elevated as compared to the remaining sites. This indicates a potential radiological health concern, suggesting a need for further investigation and potential mitigation measures to reduce exposure.

The Table 5 provided the measured activity concentrations of radionuclides ^232^Th, ^238^U, and ^40^K in Head (spring) drinking water from various sites in selected towns. It also shows the resulting annual effective dose in millisieverts per year (mSv/year) as well.

**Table 5:** Average activity concentration and annual effective dose from head (spring) drinking water, demonstrating geological and climatic influences on dose variation.

Selected Towns	Site or Station	^Average Activity concentrations (mBq/L)^			Annual effective dose (mSv/year)
		^232^Th	^238^U	^40^K	
Dessie	Mume	0.07 ± 0.06	1.05 ± 0.46	411 ± 4	1.86
	Sireminch	0.051 ± 0.015	0.97 ± 0.07	702 ± 6	3.18
	Minchitu	0.02 ± 0.03	1.35 ± 0.56	188 ± 2	0.85
Kombolcha	Station 1	0.050 ± 0.004	1.71 ± 0.16	548 ± 10	2.48
	Station 2	0.04 ± 0.03	3.1 ± 1.3	433 ± 6	1.96
Reference Values^28^		**0.05**	**0.5**	*	**1**

* ^40^K, a radionuclide that occurs naturally in a fixed ratio to stable potassium, is not included. This is because potassium is an essential element for humans and its concentration in the body is controlled by metabolic processes, BDL is for Below Detection Limit. All measured values for ^232^Th are either at or slightly above the reference value, with Minchity having the lowest and Mume having the highest concentration. ^238^U, all measured values exceed the reference value significantly, particularly in Kombolcha Station 2. All sites have annual effective doses exceeding this reference value except Minchitu site in Dessie town, indicating a higher potential radiation risk from drinking water in these locations. Sireminch in Dessie has the highest annual effective dose (3.18 mSv/year), while Minchitu in Dessie has below the recommended value.

### Discussions

The findings underscore that not only geology but also climatic factors such as precipitation, humidity, and soil moisture strongly influence radionuclide mobility. Annual rainfall enhances leaching and transport of uranium and thorium, especially along hydrological pathways like the Borkena River. Thus, both geological formations and climatic variations act together to shape the natural radioactivity levels in drinking water across Dessie and Kombolcha.

#### Natural radionuclide activity concentration

As it is able to seen from Fig. 2, the geological formation of the two towns is from Basaltic and Sedimentary mud rocks. The natural occurrences of uranium and thorium in such geological formations are 0.1–1 ppm and 0.1–4 ppm in Basaltic respectively, and 1–5 ppm and 10–13 ppm in Sedimentary Mud rocks respectively^1^.

**Fig. 3:**
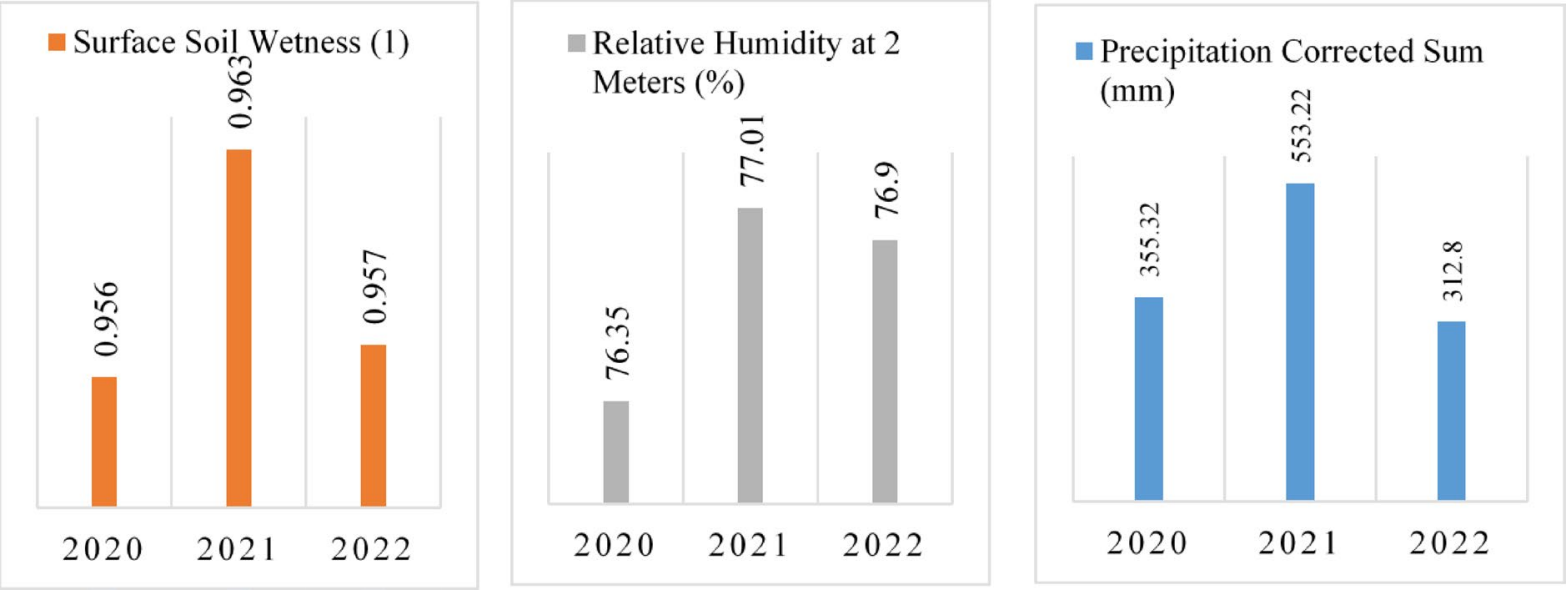
Climatic parameters (precipitation, humidity, and soil moisture) influencing the mobility of natural radionuclides in the study area (Source: NASA POWER dataset).

Surface soil moisture, humidity, and precipitation enhance radionuclide concentrations by facilitating their mobility^29^. According to NASA POWER climate data for the period 2020 to 2022, shown in Fig. 3, surface soil moisture in the study area ranges from 0.956 to 0.963, relative humidity at 2 m height ranges from 76.35 to 77.01%, and the corrected precipitation sum ranges from 312.8 to 553.22 mm. Due to the elevation gradient, downstream areas of the Borkena River may be impacted by contaminants originating in Dessie and Kombolcha. This study accounts for climatic conditions, which affect the emanation and transport of radon gas from the subsurface. As previously discussed, radon is a radioactive gas resulting from the uranium-238 decay series.

Surface soil moisture enhances the mobilities of ^238^U, ^232^Th and ^40^K in a porous soil, where moistures transport radionuclides across the soil^30,31^. Precipitation and humidity also permit the mobilities of gaseous radionuclide in the environment. These factors allow radionuclides to be leached into water at different sources^32,33^. No geographical barriers exist to impede the mobility of these radionuclides. Uranium typically exhibits higher solubility in geochemical solutions and water compared to thorium^31^; accordingly, this study found uranium activity concentrations to be higher than those of thorium in the sampled water. Comparing the two locations, Kombolcha shows higher activity concentrations than Dessie. As illustrated in Fig. 2, Dessie sits at a higher elevation, with surface and subsurface water flowing toward Kombolcha via the Borkena River. This hydrological gradient facilitates the downstream transport of radionuclides, leading to an accumulation and higher activity concentrations in Kombolcha’s water, particularly within the river system.

Pearson correlation analysis between downstream distance (within 26 km) and radionuclide activity concentrations in running water showed positive relationships for ^232^Th (r = 0.30), ^238^U (r = 0.51), and ^40^K (r = 0.49). The moderate correlations observed for ^238^U and ^40^ K suggest possible downstream enrichment resulting from the transport of radionuclide-bearing materials from the higher-altitude Dessie area toward Kombolcha.

Subsurface radionuclide mobility leads to higher activity concentrations at lower altitudes due to geochemical transport. As a result, water samples from wells, springs, and rivers in Kombolcha exhibit higher radioactivity levels than those in Dessie. This study identifies surface water transport as the main driver for the elevated activity concentrations observed at the lower-altitude site.

Both towns are located within the Ethiopian northern plateau, which is composed of igneous and sedimentary rock formations^34^. Gradual leaching of these formations by water in the study area increases the activity concentrations of naturally occurring radionuclides. Therefore, as supported by previous study^35^, the geological formations and associated geochemical conditions play a key role in the elevated activity concentrations observed in both Dessie and Kombolcha. In addition, climatic factors such as surface soil moisture, relative humidity, and precipitation enhance the mobility of natural radionuclides in accordance with the geochemical setting and topography of the area^36^. Overall, the study indicates that more soluble radionuclides tend to accumulate at lower altitudes, resulting in higher activity concentrations in Kombolcha than in Dessie. The higher activity concentration of uranium relative to thorium in water samples is attributed to its greater solubility in aqueous and geochemical environments.

This study establishes that the radiological profile of drinking water in the Ethiopian northern plateau is dictated by a synergistic relationship between lithogenic sources and hydro-climatic drivers. The significance of these findings lies in identifying the topographical gradient as a primary mechanism for radionuclide accumulation, where uranium’s high solubility leads to its preferential enrichment in lower-altitude catchment areas like Kombolcha. This indicates water safety frameworks in mountainous regions must transition from static geological assessments to dynamic hydrological models that account for rainfall-driven transport. These results provide a critical baseline for local health authorities to implement targeted filtration and monitoring strategies in downstream communities to mitigate long-term radiotoxicity risks.

#### Radiation doses from natural radionuclides

The presence of natural radionuclides in drinking water is a critical health issue because chronic low-level radiation exposure can be hazardous. Radionuclides such as uranium, thorium, and radon, along with their decay products, occur naturally in drinking water at a wide range of concentrations.

The highest annual effective dose is observed in Sireminch (3.18 mSv/year), followed by Mume (1.86 mSv/year) in Dessie, and Station 1 (2.48 mSv/year) in Kombolcha. Minchitu in Dessie town has low annual effective dose (0.85 mSv/year). All sites have ^238^U levels exceeding the reference value, indicating a potential radiological health risk due to its high radiotoxicity and ability to replace calcium in bones. Its gaseous radioactive, radon and its decay progeny cause a serious lung problem and kidney failure. Although ^40^ K is a naturally occurring isotope with relatively low radiotoxicity, its high activity concentrations could contribute significantly to the overall radiation dose. While most sites have ^232^Th levels close to or slightly above the reference value, the potential health impact is relatively lower compared to ^238^U. In general, certain sites, particularly Sireminch in Dessie and Station 1 in Kombolcha, exhibit higher annual effective doses, primarily due to elevated ^238^U and ^40^K activity concentrations. Continuous monitoring and potential mitigation measures are recommended to ensure the safety of the drinking water in these regions.

## Conclusions

This study demonstrates that natural radioactivity in drinking water is governed by the synergistic influence of geological formations and climatic conditions. The basaltic and sedimentary mudrocks of Dessie and Kombolcha serve as primary lithogenic sources, enriched in uranium, thorium, and potassium. Climatic factors specifically precipitation, humidity, and soil moisture facilitate radionuclide mobility by promoting leaching and transport into groundwater, spring sources, and river systems. The lower elevation of Kombolcha, combined with rainfall-driven runoff originating from Dessie via the Borkena River, accounts for the consistently higher activity concentrations observed downstream. However, hydrological correlation and limited number of sampling sites reduces the statistical power of the analysis, and further investigation is recommended to confirm this trend. Radiation dose assessments indicate that annual effective doses at several sites exceed international safety limits, primarily driven by elevated uranium levels, thereby posing potential long-term radiological health risks. These findings underscore the necessity of continuous monitoring and the integration of geological and climatic variables into water safety frameworks. Future research should utilize updated geological mapping and high-resolution climatic datasets to further refine the predictive modeling of radionuclide activity across Ethiopia’s northern plateau.

## Data Availability

The climate factor data are available on https://power.larc.nasa.gov/data-access-viewer/

## References

[1] A.M. Tye, A.E.M., P.L. Smedley, *Distribution of natural radioactivity in the environment*. Open Report OR/17/01, ed. S.C.P. Land. 2017, British Geological Survey.

[2] IAEA, *Guidelines for radioelement mapping using gamma ray spectrometry data*, ed. I. 1011–4289. 2003, VIENNA: AEA-TECDOC-1363.

[3] Kallio, A., Leikoski, N. & Otaki, M. Natural radioactivity of residues from groundwater treatment facilities in Finland. *J. Radiol. Prot.* **43**(3), 031517 (2023). 10.1088/1361-6498/acf8d1 37699385

[4] Zhang, B. et al. Hydrogeochemical characteristics and enrichment regularities of groundwater uranium in the Erlian basin, China. *Appl Geochem* **170**, 106094 (2024).

[5] Cho, H. et al. Water-rock interactions of uranium deposits: A field investigation and laboratory batch experiment. *Appl. Geochem.* **161**, 105880 (2024).

[6] Asikainen, M. & Kahlos, H. Natural radioactivity of drinking water in Finland. *Health Phys.* **39**(1), 77–83 (1980). 7419411 10.1097/00004032-198007000-00009

[7] Syaeful, H., Sukadana, I. & Sumaryanto, A. Radiometric mapping for naturally occurring radioactive materials (NORM) assessment in Mamuju, West Sulawesi. *Atom Indones.* **40**(1), 33–39 (2014).

[8] Hwang, J., Lee, J.-Y. & Viaroli, S. Occurrence and geochemistry of altered radioactive accessory minerals as sources of radionuclide in Mesozoic granite aquifers (Korea). *Chemosphere* **359**, 142326 (2024). 38763398 10.1016/j.chemosphere.2024.142326

[9] Sahoo, S. K. A., Tokonami, S. & Sorimachi, A. Thoron (^220^Rn) in environment and its related issues. *J Nuclear Radiochem Sci* **11**(1), 1–9 (2010).

[10] Giri, S. et al. Major ion chemistry and suitability of groundwater resources for different utilizations in mica mining areas, Jharkhand, India. *Geochem. Trans.* **26**(1), 5 (2025). 40389614 10.1186/s12932-025-00099-x PMC12090656

[11] Zandvakili, Z., Nishio, Y. & Sano, Y. Geofluid behavior prior to the 2018 Hokkaido Eastern Iburi earthquake: Insights from groundwater geochemistry. *Prog. Earth Planet. Sci.* **11**(1), 32 (2024).

[12] Yonehara, H., Aoyama, T. & Radford, E. P. Natural background radiation and radon exposure in Japan. *Health Phys.* **71**(2), 340–345 (1996).8698576

[13] Kurttio, P. et al. Renal effects of uranium in drinking water. *Environ. Health Perspect.* **110**(4), 337–342 (2002). 11940450 10.1289/ehp.02110337 PMC1240795

[14] Ademola, J. A. & Ojeniran, O. R. Radon-222 from different sources of water and the assessment of health hazard. *J. Water Health* **15**(1), 97–102 (2017). 28151443 10.2166/wh.2016.073

[15] Anderson, W. A. et al. Uranium exposure and kidney tubule biomarkers in the multi-ethnic study of atherosclerosis (MESA). *Environ. Res.* **271**, 121060 (2025). 39922262 10.1016/j.envres.2025.121060 PMC11959630

[16] Darby, S. et al. Radon in homes and risk of lung cancer: Collaborative analysis of individual data from 13 European case-control studies. *BMJ* **330**(7485), 223 (2005). 15613366 10.1136/bmj.38308.477650.63 PMC546066

[17] Appleton, J. D. Radon: Sources, health risks, and hazard mapping. *Ambio* **36**(1), 85–89 (2007). 17408197 10.1579/0044-7447(2007)36[85:rshrah]2.0.co;2

[18] Taha, T., Essia, H. & Tammam, A. A. Assessment of the annual effective dose due to intake of natural radionuclides from some food samples. *Assiut Univ. J. Multidiscip. Sci. Res.* **53**(2), 267–275 (2024).

[19] Jadiyappa, S., *Radioisotope: applications, effects, and occupational protection.* Principles and applications in nuclear engineering-radiation effects, thermal hydraulics, radionuclide migration in the environment, 2018: p. 19–47.

[20] Chauhan, A. & Jindal, T. Microbiological methods for water, soil and air analysis. In *Microbiological methods for environment, food and pharmaceutical analysis* 93–196 (Springer, Berlin, 2020).

[21] Braysher, E., *Development of reference materials and evaluation of decay data in support of characterisation of naturally occurring radioactive material*. 2021, University of Surrey.

[22] Aguiar-Amado, P. P., Amado, V. A. & Aguiar, J. C. Full-energy peak determination from total efficiency and peak-to-total ratio calculations. *Nucl. Instrum. Methods Phys. Res. A* **990**, 164980 (2021).

[23] ISO/IEC, *General requirements for the competence of testing and calibration laboratories*. 2017.

[24] IAEA, *Determination and interpretation of characteristic limits for radioactivity measurements*, in *series No. 48*, I.A.Q.i.N. applications, Editor. 2017, VIENNA.

[25] Ibikunle, S., Ajayi, O. & Dada, O. Activity concentration assessment of natural radionuclides in borehole water and it‟ s radiological impact from Akure, Nigeria. *Int. J. Sci. Res.* **4**, 2875–2879 (2013).

[26] Ibikunle, S. et al. Natural radioactivity measurement of water and sediment from the historic Ikogosi warm and cold spring, Nigeria. *Niger. J. Pure Appl. Phys.* **8**(1), 20–26 (2018).

[27] Eckerman, K. et al. ICRP publication 119: compendium of dose coefficients based on ICRP publication 60. *Ann. ICRP* **41**, 1–130 (2012). 23025851 10.1016/j.icrp.2012.06.038

[28] Joint, F. and W.H. Organization, *criteria for radionuclide activity concentrations for food and drinking water*. 2016, Joint FAO/IAEA division of nuclear techniques in food and agriculture

[29] Gee, G., Rai, D. & Serne, R. Mobility of radionuclides in soil. *Chem Mob React Soil Syst* **11**, 203–227 (1983).

[30] Wei, X. et al. Phosphate and illite colloid pose a synergistic risk of enhanced uranium transport in groundwater: A challenge for phosphate immobilization remediation of uranium contaminated environmental water. *Water Res.* **255**, 121514 (2024). 38554633 10.1016/j.watres.2024.121514

[31] Ali, K. et al. Assessment of radioactive substance transfer and its ecological and health impacts on the Nasser Lake ecosystem. *Sci. Rep.* **15**(1), 26115 (2025). 40681563 10.1038/s41598-025-09342-y PMC12274626

[32] Ahmed, G. E. & Zeleke, H. G. Radon gas mapping for environmental assessment in Dessie, Ethiopia. *Sci. Rep.* **15**(1), 37271 (2025). 41136497 10.1038/s41598-025-21201-4 PMC12552722

[33] Nam, Y. et al. Radon concentration and affecting environmental conditions in water-curtain heated cultivation facilities. *Heliyon*10.1016/j.heliyon.2024.e30563 (2024). 38742076 10.1016/j.heliyon.2024.e30563 PMC11089361

[34] Tzortzis, M. & Tsertos, H. Determination of thorium, uranium and potassium elemental concentrations in surface soils in Cyprus. *J. Environ. Radioact.* **77**(3), 325–338 (2004). 15381324 10.1016/j.jenvrad.2004.03.014

[35] Tye, A., A. Milodowski, and P. Smedley, *Distribution of natural radioactivity in the environment.* 2017.

[36] Payne, T. E. & Edis, R. Mobility of radionuclides in tropical soils and groundwater. In *Radioactivity in the environment* 93–120 (Elsevier, Amsterdam, 2012).

